# Supplementary material for: Relating natural image statistics to patterns of response covariability in macaque primary visual cortex
Source: Nat Commun. 2025 Jul 22;16:6757. doi: 10.1038/s41467-025-62086-1 (PMC12284261; doi:10.1038/s41467-025-62086-1)
Supplement: Supplementary file 1 — Supplementary Information [file 41467_2025_62086_MOESM1_ESM.pdf]

Supplementary Information

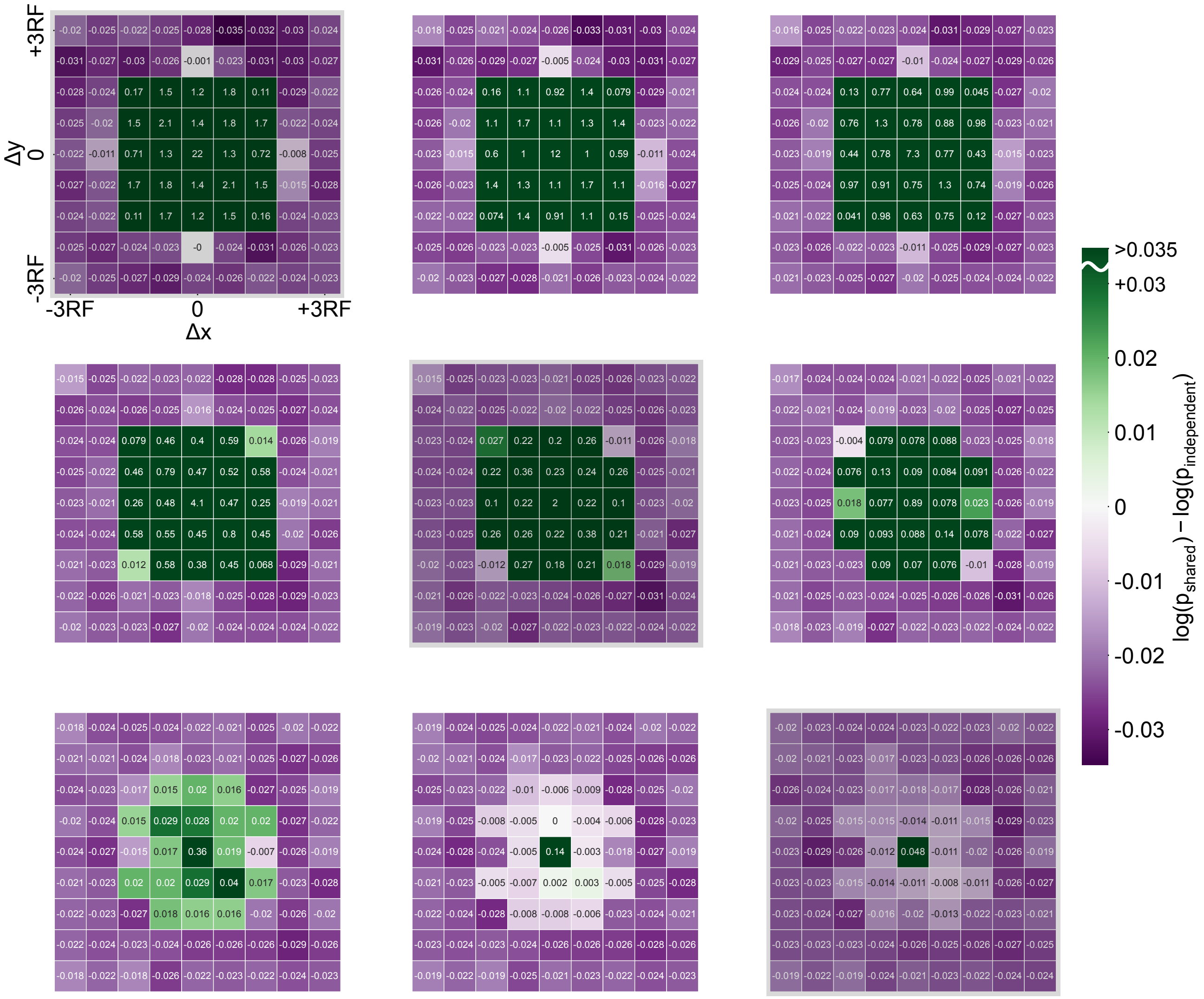

Supplementary Figure 1. Evaluating shared versus independent GSM models on natural image statistics across all orientation preferences. The graphs compare the log-likelihoods of natural images analyzed by shared versus independent GSM models, whose parameters (i.e., covariance matrices) are optimized using 10,000 natural and white noise images (see Methods). The log-likelihood ratio over a different subset of 10,000 test images determines which pairwise GSM model better captures the statistics of natural images. 2D maps illustrate the likelihood across different orientation preferences (from $\Delta\theta = 10$, top left, to $\Delta\theta = 90$, bottom right). The position of each entry in the map indicates the relative position of the filters of the two neurons. The color map for positive values (green, indicating that shared GSM is better) is adjusted to the same range as negative values to improve visualization; the actual numerical values are reported in the corresponding bins. When the receptive fields overlap, the ratio is distinctly positive, suggesting that the shared GSM model exhibits a higher likelihood than the independent GSM model. In contrast, with non-overlapping receptive fields, the ratio turns mostly negative, depending on orientation preferences, suggesting that the independent GSM model more effectively captures image statistics. The three overlaid gray boxes align with the example 2D maps depicted in Fig. 2B.

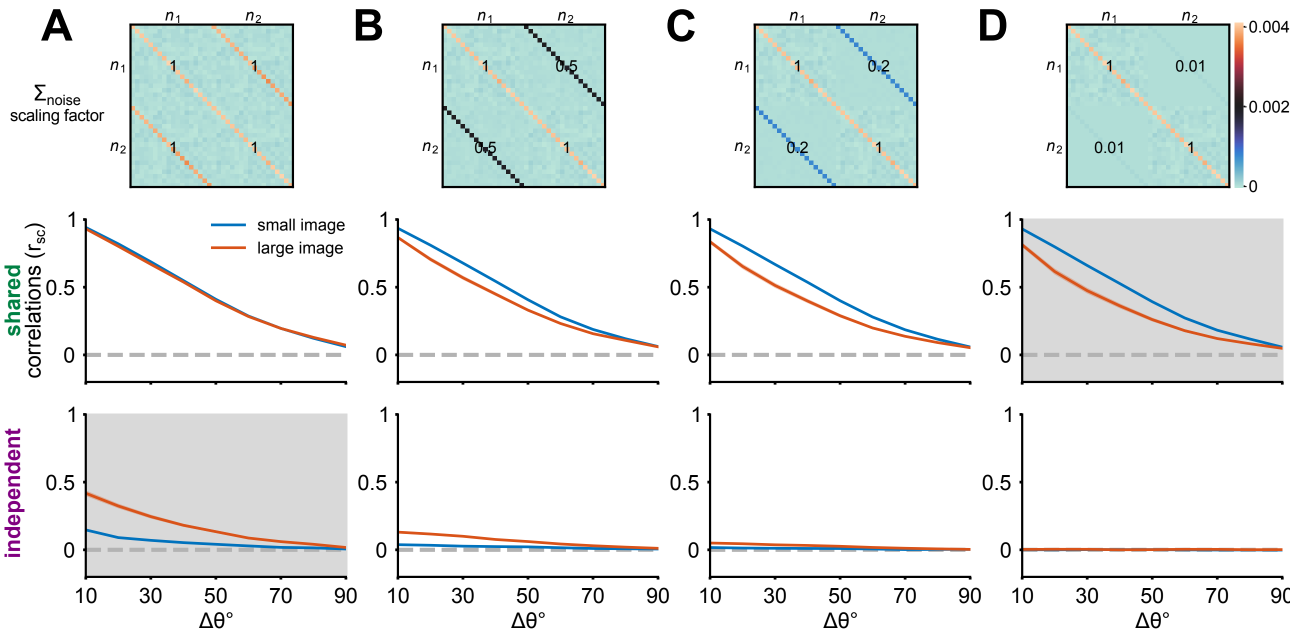

Supplementary Figure 2. The level of shared additive noise influences the magnitude of correlations in the pair-wise GSM models. The top row shows covariance matrices between the filters of a pair of neurons with similar orientation preference ($\Delta\theta = 10$), for the shared additive noise ($\boldsymbol{\eta}$). The leftmost panel is the covariance learned from white noise images. In the examples in **B-D** the level of shared additive noise was adjusted. The numbers written in the covariance matrix indicate the scaling factor we applied to the entries in the corresponding block of the matrix. Since the covariability between a pair of neurons stems from two sources—mixer (global modulator) and input noise—reducing the impact of one leads to the dominance of the other. In the shared model (middle row), decreasing the additive noise (from **A** to **D**) results in a larger relative contribution of the shared global modulator to correlated variability, and therefore the modulation by surround stimuli becomes more evident (because the effect of additive noise is stimulus independent). In the independent model (bottom row) decreasing the additive noise results in a larger relative contribution of the independent global modulators to independent variability. Therefore the modulation of r_{sc} by surround stimuli is evident only when the additive noise level is sufficiently high to produce correlated variability. The overlaid gray boxes align with the results depicted in Fig. 3.

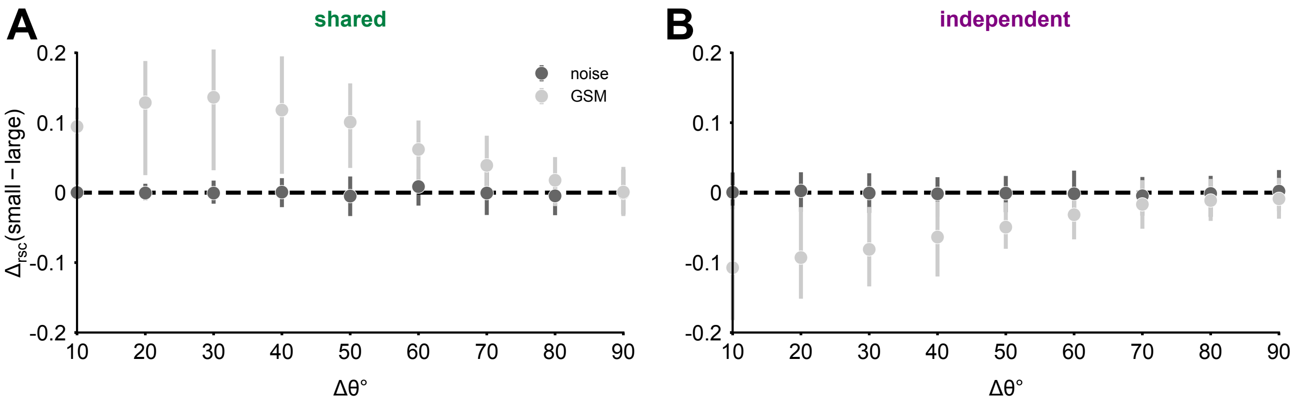

Supplementary Figure 3. Sampling only from input noise leads to no modulation of correlation, as expected. Setting the global modulator to zero configures the inference to rely on input noise (refer to the brown box in Fig. 3B). Each solid circle represents the average of Δr_{sc} across 200 natural images, and the error bars represent interquartile ranges. (**A**) Modulation of correlations for small and large images for the shared GSM model. In the model with only input noise, the correlations remains similar for both small and large images, with a resultant Δr_{rsc} of zero across all orientation preferences (dark gray circles). This is aligned with the schematic of the contribution of the global modulator and input noise on correlations: identical brown boxes that yield the same contribution in both small and large images. The shared GSM model shows suppression of correlations (light gray circles). (**B**) Same as A, but for the independent GSM model. The dark gray circles indicate no modulation of correlations due solely to input noise, while the light gray circles depict facilitation of correlations.

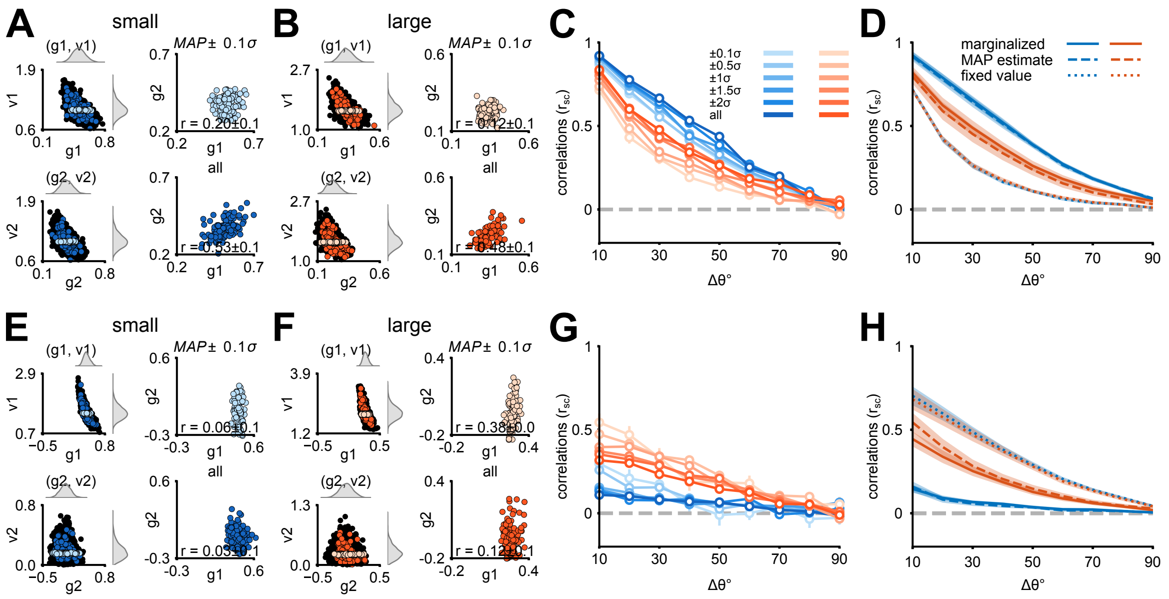

Supplementary Figure 4. Comparing marginalization, maximum a posteriori (MAP) estimation, and fixed global modulator values. This analysis contrasts three approaches for handling the global scaling variable: (1) marginalizing it, (2) using the MAP estimate, and (3) fixing it at a constant (image-independent) value. The top row of the figure presents results for the shared modulator model, while the bottom row illustrates the independent modulator model. In panels (**A-B**) and (**E-F**), the left panels show, for a single image, samples from the joint posterior of g_1 and v_1 (top) and g_2 and v_2 (bottom). In the shared model, v_1 and v_2 are identical due to the single global modulator. A key observation (right panels) is the noticeable difference in correlation when comparing samples near the MAP estimate of v (top) to those obtained by marginalizing v (labeled "all"; bottom). Panels (**C**) and (**G**) systematically vary interval sizes around the MAP estimate to measure correlations across corresponding samples, for multiple images driving the centered filter. While changes in interval size affect correlations as predicted by the effects of marginalization, the qualitative differences between small and large intervals remain consistent. To investigate further, panels (**D**) and (**H**) replace interval-based sampling with a direct comparison of posterior distributions. Specifically, we first capture the posterior distribution of the global modulator for each test image, then compare results obtained by replacing marginalization over the global modulator with its MAP estimate. We also test a condition where the global modulator is fixed at a value of 1. Results indicate that MAP estimation approximates marginalization, capturing the overall trend despite being a simplified representation of the full distribution. This occurs because MAP estimates differ in magnitude between small and large images, leading to suppression or facilitation of correlations, depending on whether the global modulator induces shared or independent variability. Conversely, fixing the global modulator at a constant value eliminates differences in correlation between small and large images, as expected. In summary, while the MAP estimate can recover opposite contextual effects of correlation, the magnitude of correlations further depends on marginalization over the global modulator, highlighting its role in accurately capturing variability.

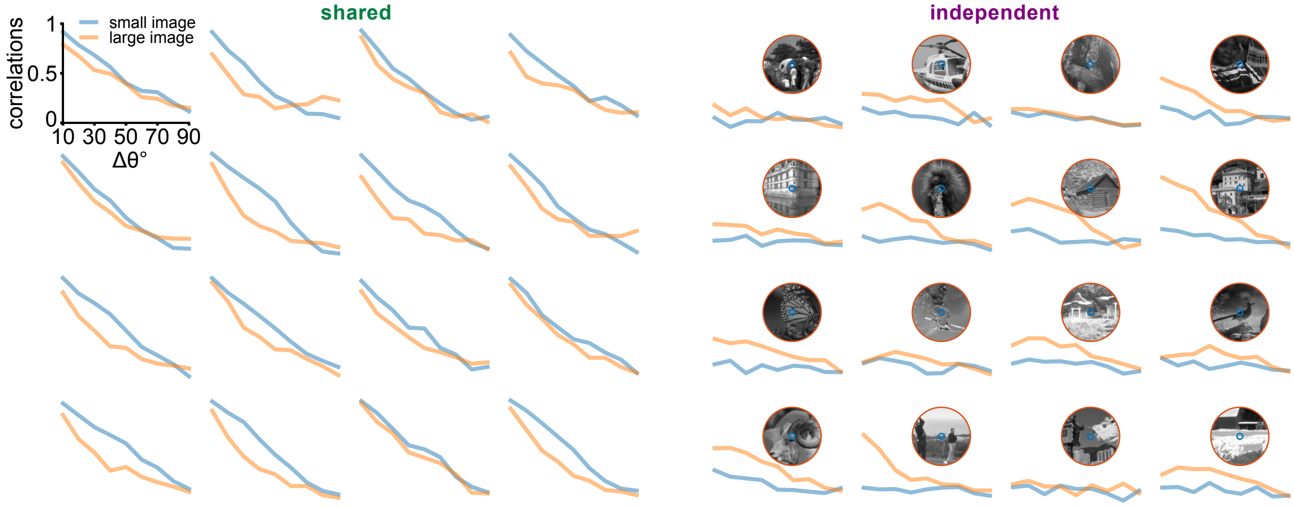

Supplementary Figure 5. The modulation of correlations in pairwise GSM models varies from one image to another. Each panel shows correlations r_{sc} for an example natural image, using the same conventions as main Fig. 3. Given the natural image statistics associated with shared and independent models for different configurations of model neurons (Fig. 2), this figure shows r_{sc} for pairs with overlapping RFs for the shared model, and pairs with non-overlapping RFs (separated by about 2x the RF size) for the independent model.

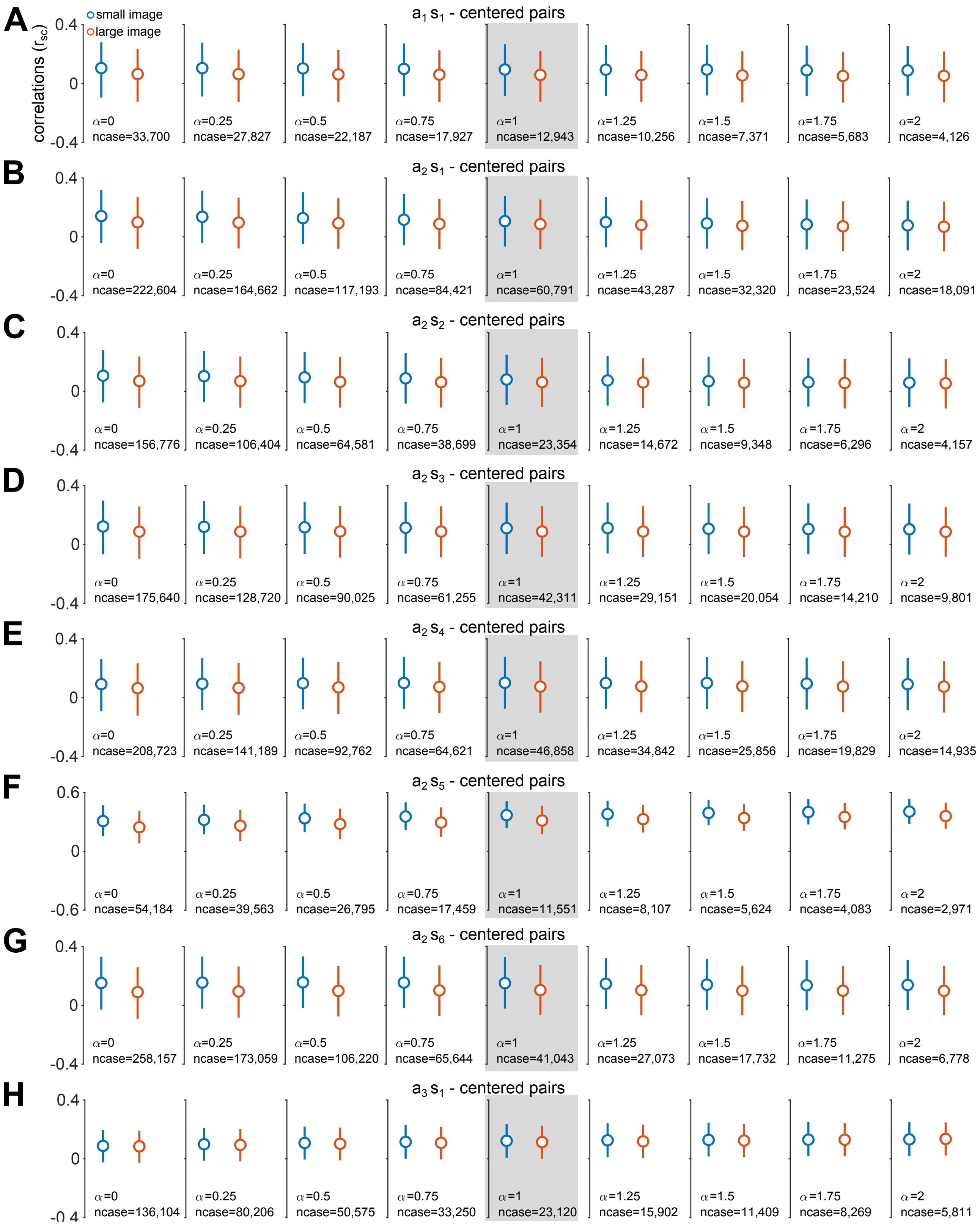

Supplementary Figure 6. Correlations modulation in V1 remained consistent despite variations in the inclusion criteria for centered pairs (Utah array data). (**A-H**) Inclusion criteria involved comparing the stimulus-driven responses to the mean plus a scaled standard deviation of spontaneous activity. We modified the scaling factor applied to the standard deviation of spontaneous activity (denoted as 'α' in each panel). Each circle represents the average of correlations distributions, and the error bars span the 25th to 75th percentiles of the samples. The value of "ncase" indicates the total number of cases (neural pairs and images) included for a given scaling factor. Each row is a different recording session (**A-G** from Coen-Cagli (2015)). The superimposed gray boxes denote the scaling factors used in the main figures (Fig. 4 and 5). In all sessions, the results demonstrated a consistent suppression trend in correlations across various scaling factors.

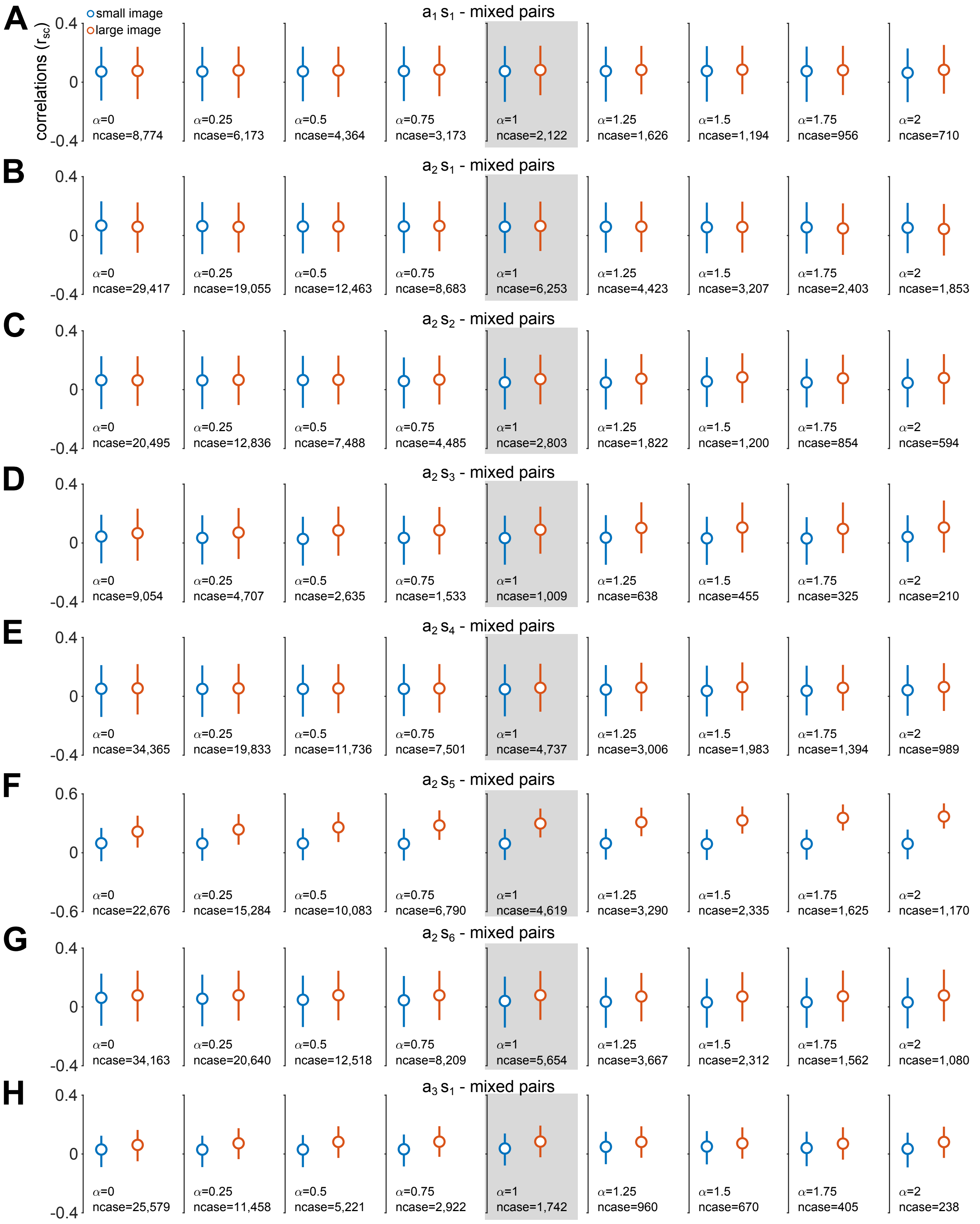

Supplementary Figure 7. Correlations modulation remained consistent despite variations in the inclusion criteria for mixed pairs (Utah array data). (**A-H**) Same conventions as in Supplementary Fig. 8. In all sessions, the results demonstrated a consistent facilitation trend in correlations across various scaling factors.

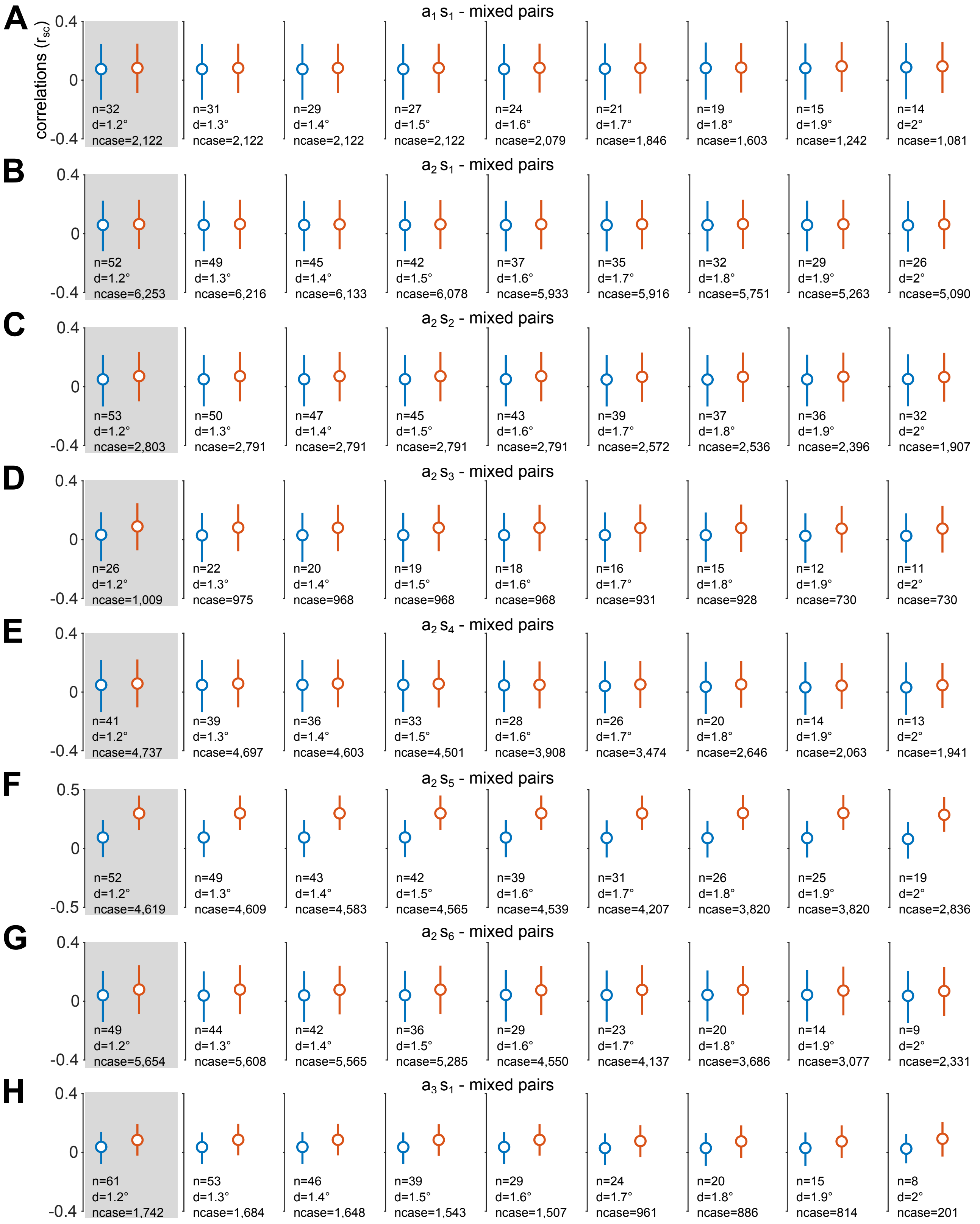

Supplementary Figure 8. Correlations modulation remained consistent despite variations in the position criterion for off-center cells (Utah array data). (**A-H**) We included off-center neurons if their RF's distance from the stimulus center was above a threshold value denoted as d (see Methods). Here we vary this threshold from 1.2° to 2° (specific values are reported in each panel). The value of 'n' reported in each panel indicates the total number of neurons included for a given threshold. All other conventions are as in Supplementary Fig. 8. In all sessions, there was a facilitation in correlations.

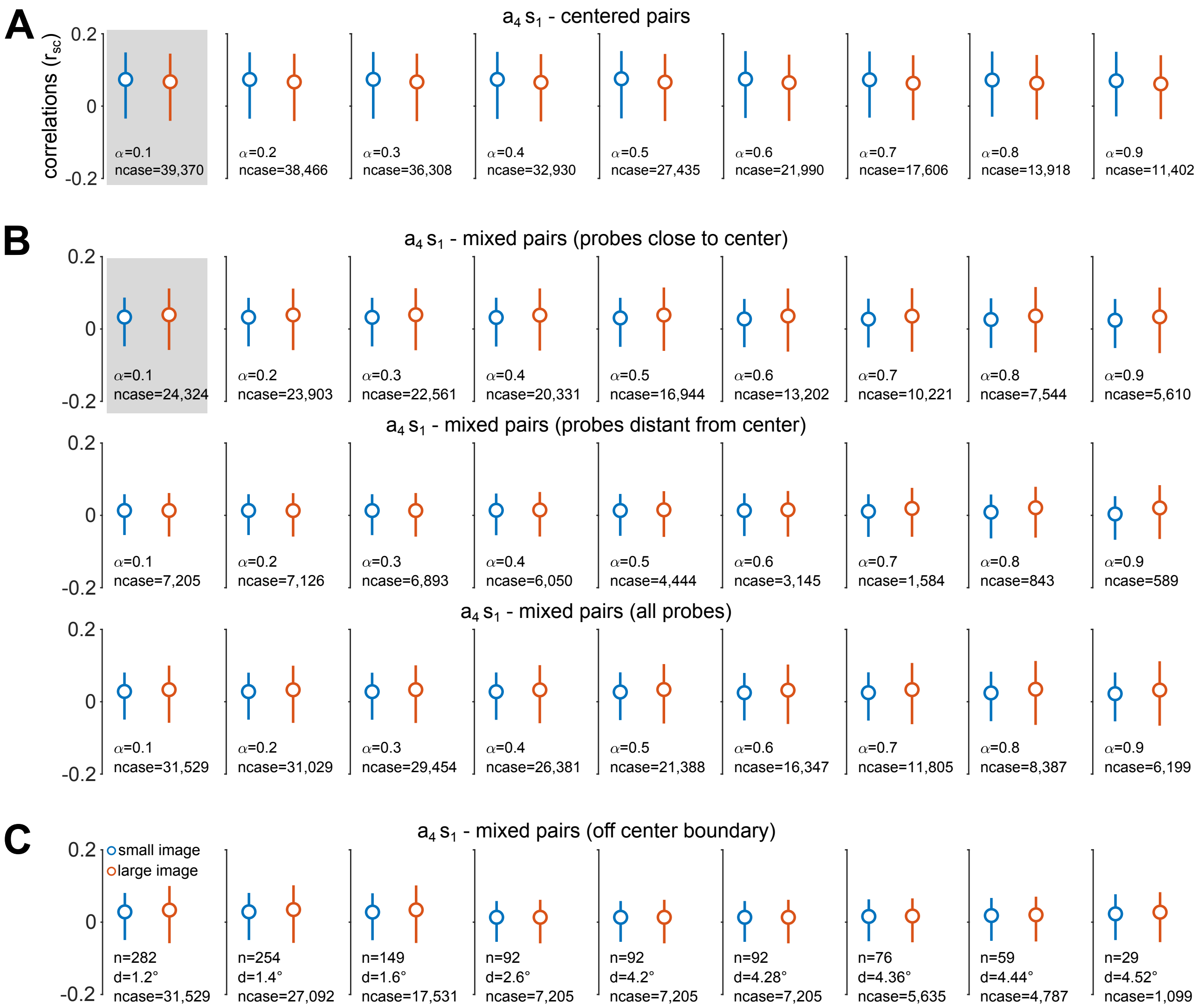

Supplementary Figure 9. Correlations modulation remained consistent despite variations in inclusion criteria (Neuropixel data). We adjusted the scaling of the standard deviation of spontaneous activity and the boundary for including off-center cells in the NeuroPixel recordings. This procedure mirrors that of Supplementary Figs. 6, 7, and 8, differing only in the specific values of the scaling factors as explained in Methods, and larger range of distance thresholds due to the recording from multiple probes. Each circle represents the average of correlations distributions, and the error bars span the 25th to 75th percentiles of the samples. (**A**) displays the modulation of correlations for centered pairs. (**B**) given that the four probes were recorded simultaneously—two near and two far from the stimulus center—we divided the results into two groups: close (probes 3 and 4) and distant (probes 1 and 2), as shown in the second and third rows of Panel B. (**C**) illustrates the results for mixed pairs while adjusting the boundary for including off-center cells, revealing a drop in the range of correlations corresponding to the results from close and far off-center cells. The superimposed gray boxes denote the scaling factors used in the main figures.

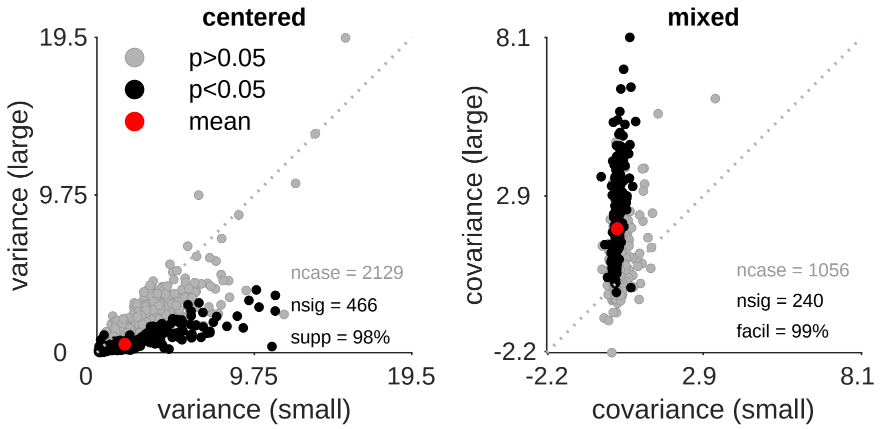

Supplementary Figure 10. Variance and covariance of population-average activity consistent with contextual modulation of correlations. For each trial, we calculated the average responses of responsive neurons separately for centered neurons and for off-centered neurons, yielding two vectors with length equal to the number of trials, representing the population-averaged activity of centered and off-centered neurons. From these, we estimated a 2×2 covariance matrix across trials. For centered neurons, we compared the variances of their population-average activity between small and large images. For the mixed group (centered and off-centered neurons), we analyzed the covariance between their population-average activities across image sizes. Notably, the variances of centered and off-centered neurons change in opposite directions, hence the effects would largely cancel out if population averages were computed by pooling all centered and off-centered neurons together. By focusing on the interaction between the groups via covariance, we avoided this bias. To assess the significance of these differences, we performed Levene's test for variance differences in the centered group and a z-test for covariance differences in the mixed group. Each circle represents one image pair from a recording session. The results show that changes in correlations align with changes in the variance and covariance of population-average activity (centered: 2129 cases, 466 significant, 98% of significant cases showed suppression; mixed: 1056 cases, 240 significant, 99% of significant cases showed facilitation), effectively predicting the contextual modulation effects observed.

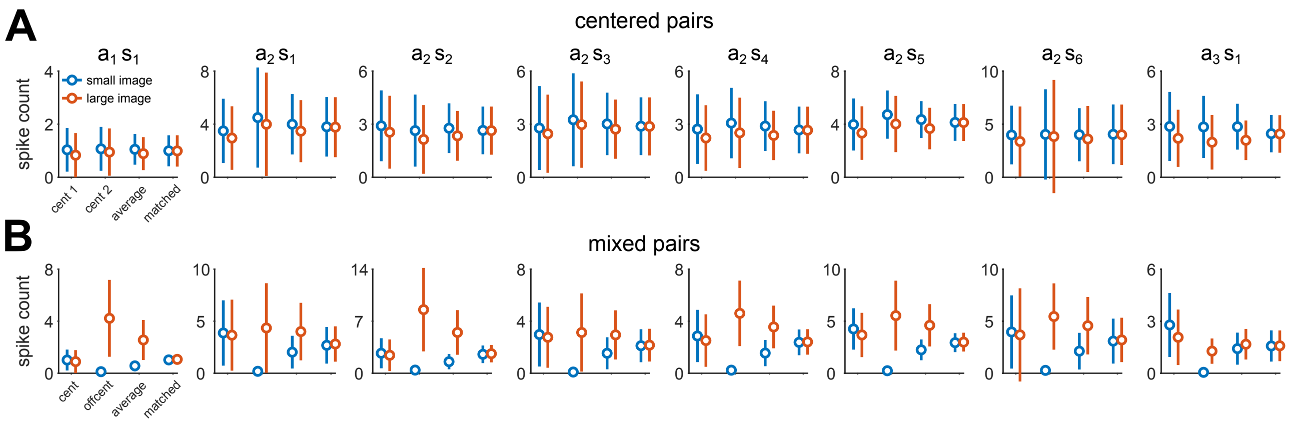

Supplementary Figure 11. Changes in firing rate for small versus large images, in single neurons and pairs. (**A**) in centered pairs, adding spatial context suppresses firing rates in both centered neurons (labeled 'cent 1' and 'cent 2' on the abscissa), and in their average (labeled 'average'). Applying mean-matched analysis to the firing rates of small and large images removes this suppression effect (labeled 'matched'). (**B**) Similar to Panel A, but for mixed pairs: there is a suppression of firing rates for the centered neuron in the pair (labeled 'cent') and a facilitation of firing rates for the off-center neuron (labeled 'offcent'). Averaging across the pair (labeled 'average') also shows facilitation, although weaker than for the off-center neuron. Applying mean-matched analysis to the firing rates of small and large images removes the facilitation effect. All sessions used Utah array recordings, with the first seven sessions' data referenced as Coen-Cagli (2015).

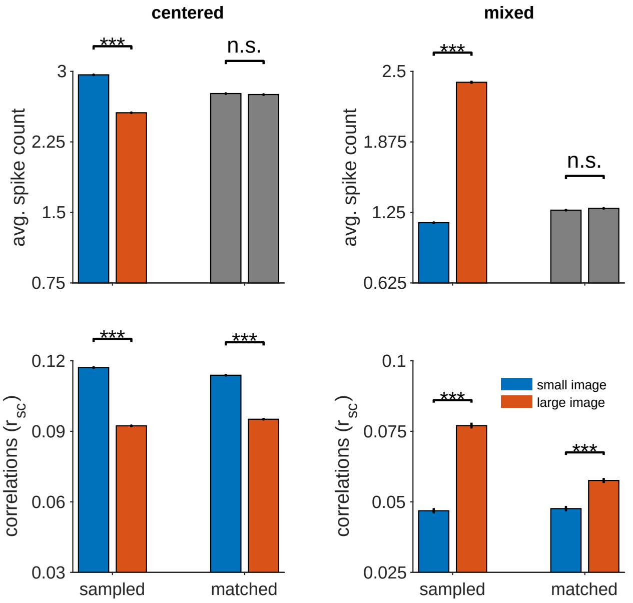

Supplementary Figure 12. Effect of mean-matching on correlations estimate. Spiking activity can directly influence estimates of correlations (De La Rocha et al., 2007; Cohen and Kohn, 2011; Schulz et al., 2015). To eliminate the potential confounding effect of changes in spiking activity due to image size on the correlation measurements, we performed a mean-matching analysis. Specifically, we examined how correlations are modulated by aperture size, both with and without mean-matching of spiking activity. In brief, for each pair of neurons, we calculated the average spike count across trials for each image and size condition. We then constructed histograms of these neural-pair-averaged mean responses separately for small and large images. To create mean-matched histograms, we subsampled the data by selecting the minimum number of samples per bin across conditions, ensuring that the distributions of average spike counts were matched between small and large images (Top panels, gray bars; see Methods for details). From these mean-matched samples, we computed the corresponding correlations. For comparison, we also randomly sampled from the raw distributions using the same number of samples as in the mean-matched condition. The results show that the modulation of correlations with aperture size is preserved both with and without mean-matching (two-sided t-test against the null hypothesis of no difference: p-value < 0.001; n.s. (not significant)), which indicates that changes in spiking activity due to image size do not account for the observed effects on correlations. Error bars represent standard error. Data from all sessions are aggregated.

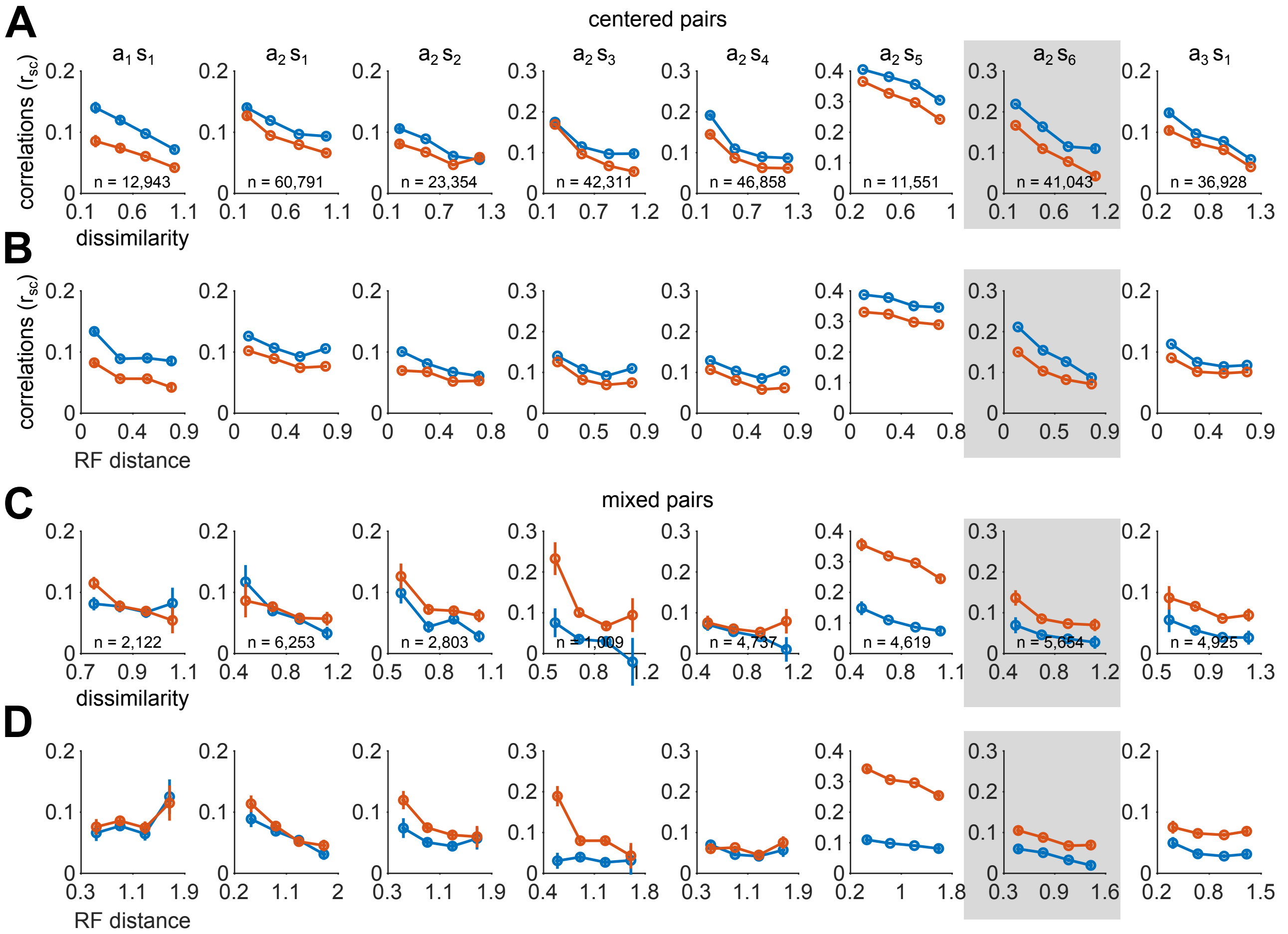

Supplementary Figure 13. The modulation of correlations by tuning dissimilarity and the RF distance between pairs of neurons is consistent across all sessions. (**A**) In centered pairs, correlations are plotted against tuning dissimilarity (1 - r_{signal}). tuning dissimilarity are divided into equal-sized intervals. Blue and orange circles represent the mean correlations for small and large images, respectively. Error bars indicate standard errors. The total number of cases across neurons and natural images is displayed at the bottom. The positions of the blue and orange circles on the x-axis correspond to the mean tuning dissimilarity for each bin, with horizontal error bars representing their respective standard errors. (**B**) Correlations are plotted in relation to the RF distance between neurons, for the same pairs as in (**A**). The mean correlation for small images consistently exceeds that for large images across all intervals. (**C, D**) Similar to (**A, B**) but for mixed pairs. Here, the mean correlation for small images is consistently lower than that for large images. In session 8, small images were deliberately centered on a human-labeled contour. To ensure appropriate neuron pair selection, we applied additional criteria: selecting neurons with a surround suppression of firing rate of more than 25% (centered) or no surround suppression (off-center). The scaling factor of the standard deviation for spontaneous activity (see Supplementary Figs. 6 and 7) was set at 1 for all sessions, except session 8, where it was reduced to 0.1 due to the lower firing rate range observed in that session. The superimposed gray boxes denote the panels used in figure 5.

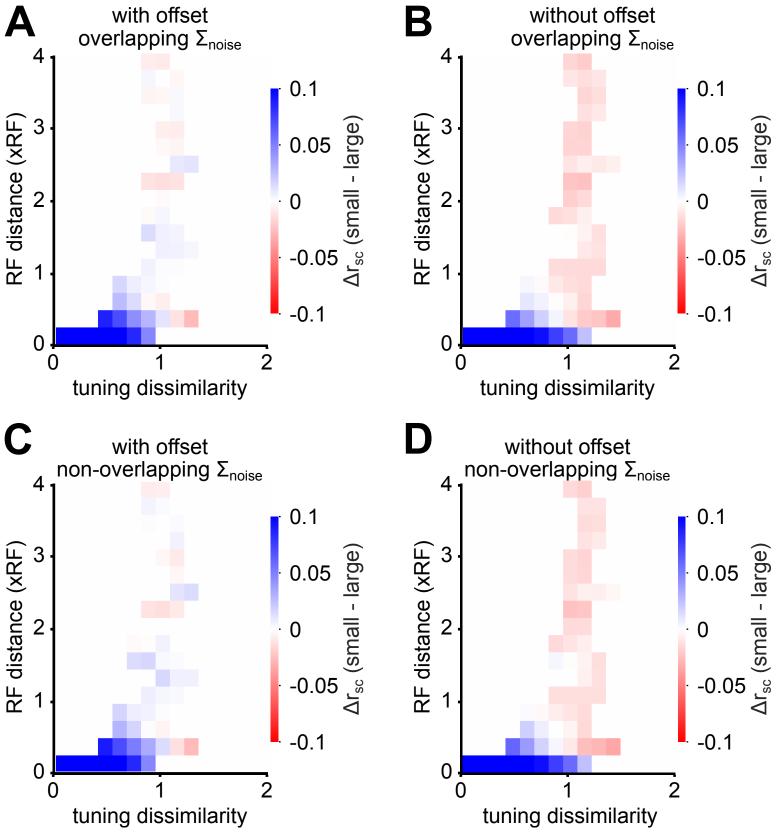

Supplementary Figure 14. Shared model alone cannot explain shifts in correlation patterns. (**A-D**) In the shared model, we applied the same conventions used in Supplementary Fig. 4 to analyze the effects of introducing an offset to the samples of gs and adding shared noise on the modulation of correlations. In filters that do not overlap, the shared GSM model exhibits negligible modulation when an offset is added and maintains a constant, small degree of facilitation without offset. This facilitation is attributed to the clipping of negative values, especially in small images. Given that the scaling factor of the noise covariance matrix in the shared model is 0.01, no differences are observed between rows. These results indicate that the shared model alone is insufficient to account for the observed transitions between suppression and facilitation in correlations within the data.

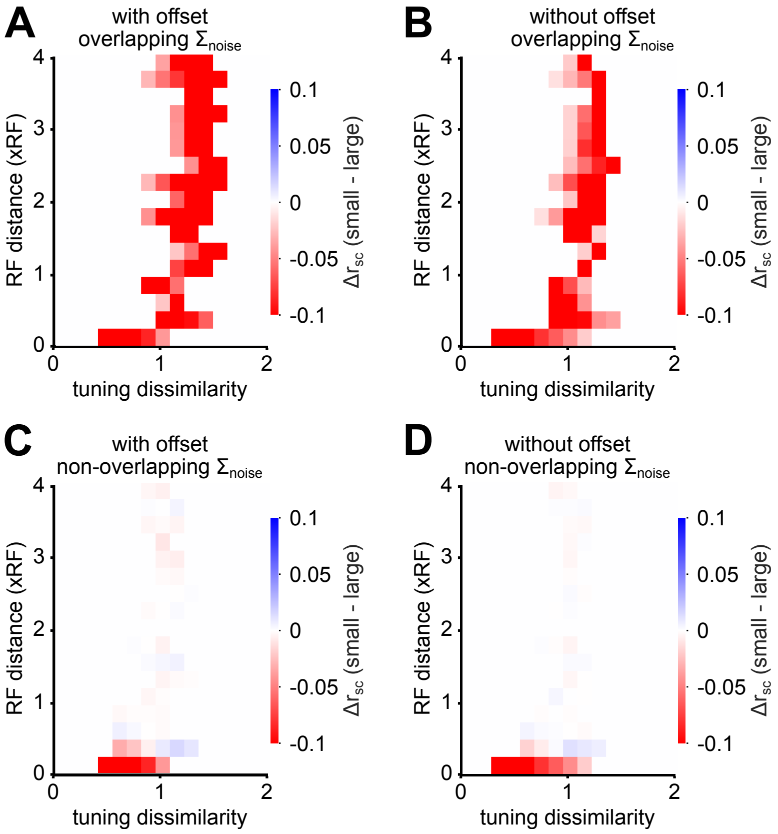

Supplementary Figure 15. Independent model alone cannot explain shifts in correlation patterns. (**A-D**) In the independent model, we applied the same conventions used in Supplementary Fig. 4 to analyze the effects of introducing an offset to the samples of gs and adding shared noise on the modulation of correlations. In cases where Σ_{noise} overlaps, the independent GSM model demonstrates facilitation of correlations, attributable to shared additive noise with a scaling factor of 1. In contrast, when Σ_{noise} does not overlap, the facilitation occurs only when filters overlap; otherwise, it remains negligible. These findings suggest that the independent model alone is insufficient to account for the observed transitions between suppression and facilitation in correlations within the data.

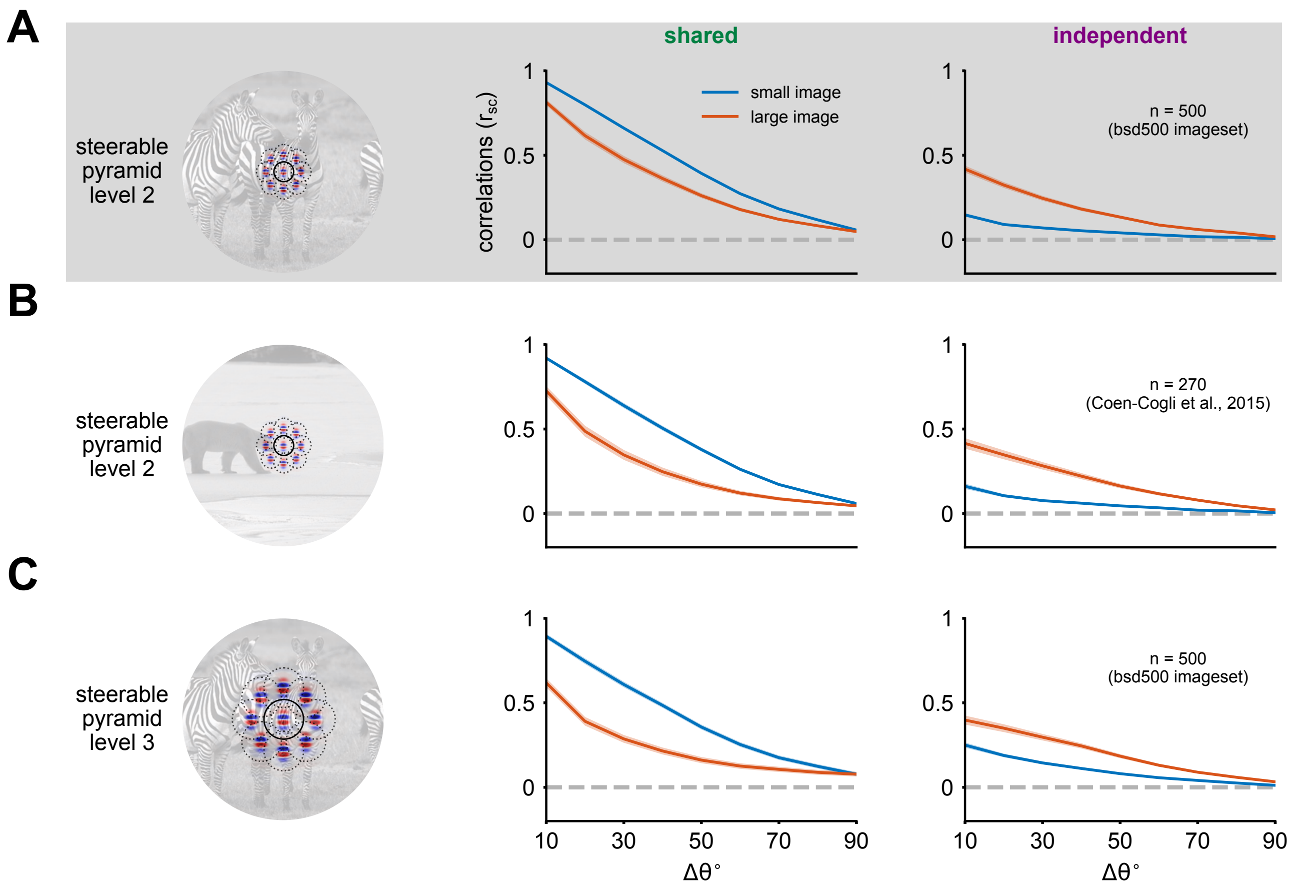

Supplementary Figure 16. The qualitative modulation of correlations remains stable across different filter sizes and image sets. (**A**) Displays these modulations for shared and independent models using the BSD500 image set and Steerable Pyramid level 2. (**B**) Explores the effects using 270 natural images from Coen-Cagli (2015), at the same pyramid level. (**C**) Examines changes using the BSD500 image set at Steerable Pyramid level 3. All other parameters are as in the main Fig. 3D. The overlaid gray box aligns with the results depicted in Fig. 3.

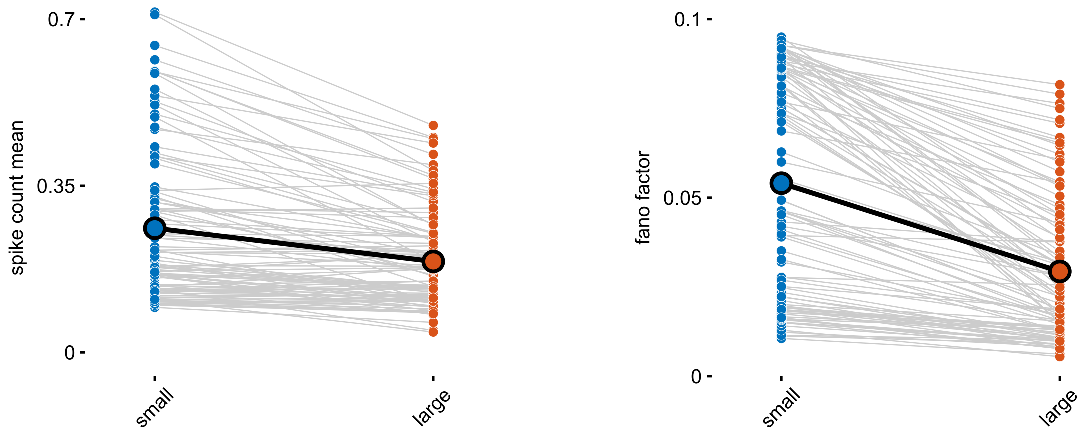

Supplementary Figure 17. Single-neuron properties in a single GSM model neuron. We analyzed a centered neuron's mean spike count and Fano factor for small and large images. Only the top 20% of images with the highest center filter responses were included, excluding non-responsive images. These results align with previous studies, demonstrating that our model preserves key single-neuron properties observed experimentally. The analyzed single neuron is representative of the neuronal population.

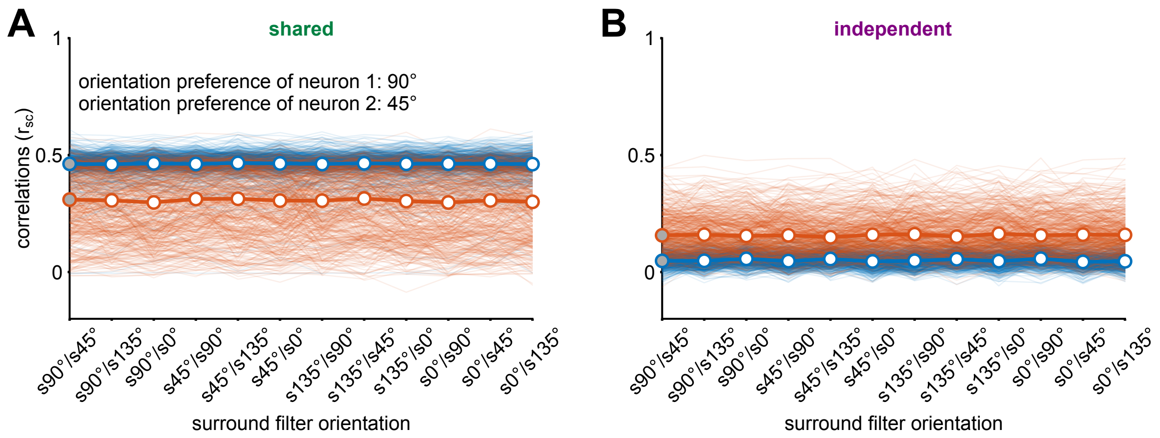

Supplementary Figure 18. Effect of surround filter orientation on correlations modulation. (**A-B**) Changing the orientation of surround filters alters the level of correlations modulation for individual natural images (thin lines) due to their distinct structures. However, when the modulation is averaged across multiple natural images, the overall level of modulation remains similar (thick lines and circles). The orientations of the two neurons are specified as 'orientation 1/orientation 2,' where the first orientation corresponds to neuron 1 and the second to neuron 2. The filled gray circles correspond to the configuration used in Fig. 3G of the manuscript.

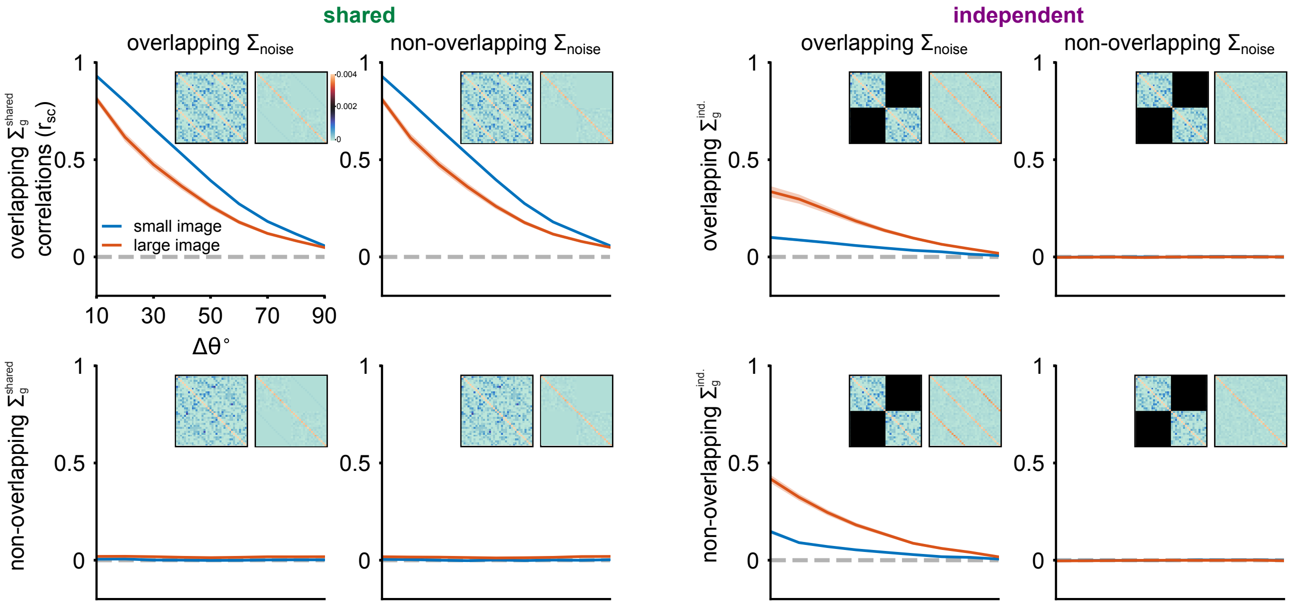

Supplementary Figure 19. This analysis compares how correlation is modulated in overlapping and non-overlapping pairs. Top row: all panels use the trained covariance matrix of the latent features (Σ_g^{shared} and $\Sigma_g^{\text{independent}}$) of overlapping pairs. In the shared model, surround stimulation modulates the correlation regardless if the covariance matrix of the additive noise (Σ_{noise}) was obtained from overlapping or non-overlapping pairs. In the independent model, using the Σ_{noise} from non-overlapping pairs leads to zero correlations for all pairs of neurons, therefore surround modulation is no longer visible. As illustrated in Supplementary Fig. 2, for maximum modulation, the scale of shared additive noise is set at 0.01 in the shared model and at 1 in the independent model. Bottom row: all panels use the trained covariance matrix of the latent features of non-overlapping pairs. In this case, for the shared model, correlations are close to zero regardless if the covariance matrix of the additive noise was obtained from overlapping or non-overlapping pairs. For the independent model, increasing the scale of shared additive noise leads to similar surround modulation as in the top row. When no source of covariance is present, i.e. with both covariance matrices from non-overlapping pairs, the correlation consistently remains zero.

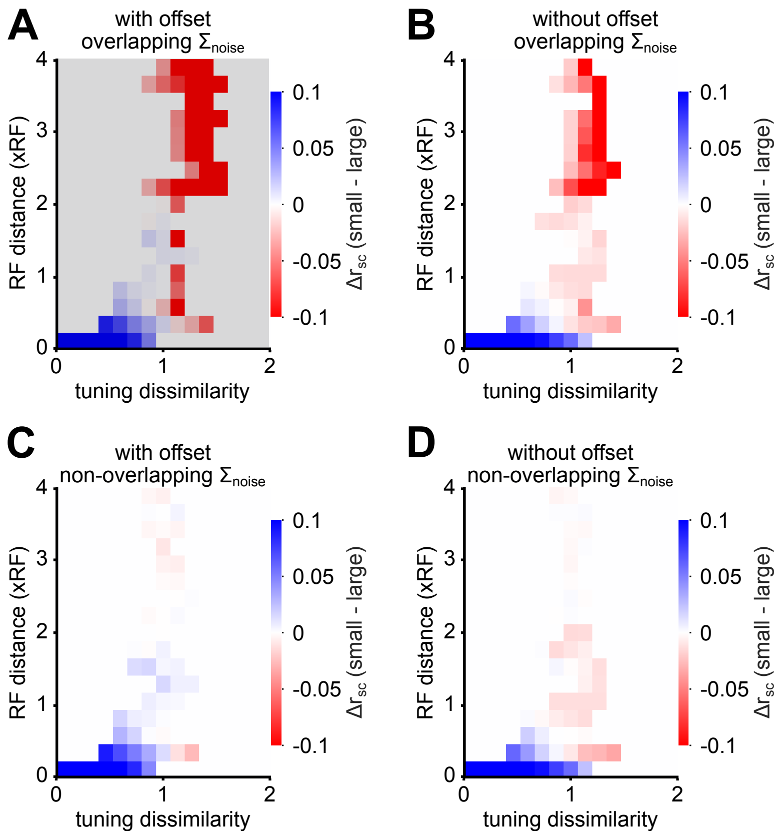

Supplementary Figure 20. Effects of adding an offset to the samples of gs and of introducing shared additive noise, on the modulation of correlations. Rectifying the values of gs when converting to neural activity (Eq. 3) can lead to clipping of negative values. This effect is more pronounced in mixed pairs, where the distant neurons typically do not respond to small images, producing negative samples near zero. To mitigate this clipping effect, especially for small images, we introduce an offset to samples of g prior to its conversion to spike counts. The two columns compare surround modulation of correlations with versus without the offset. Additionally, the two rows compare the impact of using shared additive noise with covariance Σ_{noise} estimated from overlapping filters (top) or from non-overlapping filters (bottom). (**A**) This is the condition considered in main Fig. 6. (**B**) Without offsetting g, correlations for small images are reduced, thereby producing in general more facilitation of correlations (red). (**C-D**) When using Σ_{noise} from non-overlapping filters, the modulation of correlations is less pronounced than with Σ_{noise} from overlapping filters, particularly for distant pairs. This is expected because for distant pairs, both the Σ_g and Σ_{noise} matrices are obtained from non-overlapping filter sets, therefore there is no strong source of correlated variability. Furthermore in (**D**), removing the offset to Σ_g leads to greater facilitation of correlation for distant pairs, primarily due to the clipping of negative values. As an additional control, in Supplementary Figs. 14 and 15 we measured the surround modulation of correlations in the shared and independent models.

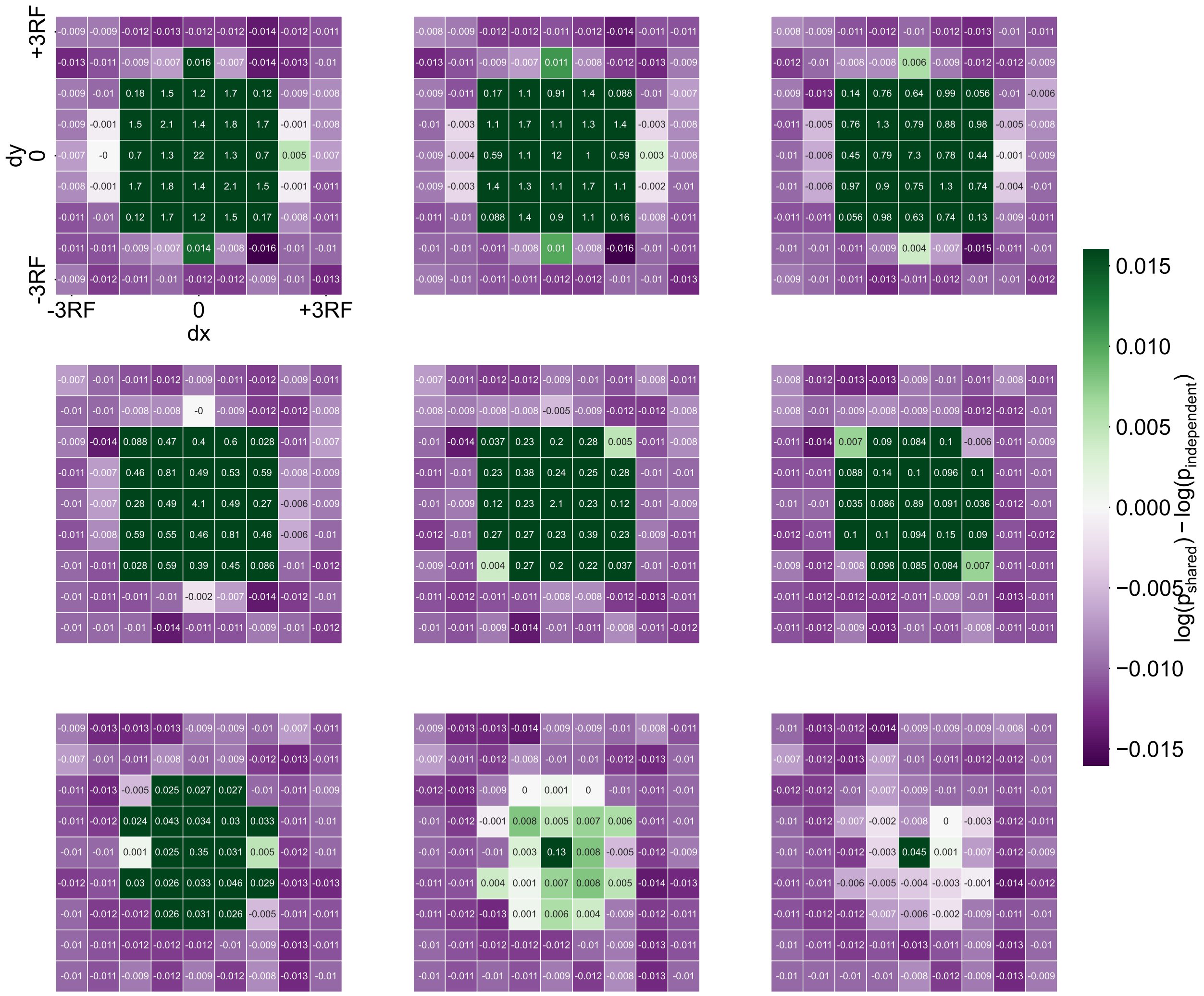

Supplementary Figure 21. Estimating prior covariance matrices using Maximum Likelihood Estimation (MLE) yields log-likelihood patterns for natural images that are highly similar to those obtained with moment-matching. Slight discrepancies between MLE and moment-matching results (Supplementary Fig. 1) may arise from differences in initialization and optimization criteria; however, these differences are minimal and do not affect the primary conclusions regarding model comparisons.

Supplementary Table 1. Details of the nine recording sessions across four animals. Note that there are 6 sessions from a single animal, a2; however, each session involves different visual inputs and neurons. First, half of those sessions are obtained from an array implanted in the right hemisphere of V1, the other half from the left hemisphere, hence these are entirely different neurons. Second, in each session from the same hemisphere, we placed the stimuli in a different location of the visual field, such that approximately one fourth of the neurons had RFs centered on the stimuli and therefore were driven by the small stimuli (see Fig. 4A top-left for one example session). This means that 1) each neuron received different visual inputs across sessions even if the same image appeared on the screen; 2) in each session, a different subpopulation was driven by the small stimuli and therefore labeled as "centered neurons". Statistical comparisons of noise correlations between small and large images were performed using two-sided t-tests across sessions. Exact p-values are reported in the table.

animal	session	recording type	cent. neuron	off-cent. neuron	cortical layer	Cohen's d (cent.)	Cohen's d (mix.)	p-val (cent.)	p-val (mix.)
a_1	s_1	Utah Array	25	32	L2/3 or 4B	0.16	-0.03	3.7×10^{-18}	0.3
a_2	s_1	Utah Array	45	52	L2/3 or 4B	0.12	-0.02	7.5×10^{-19}	0.2
a_2	s_2	Utah Array	39	53	L2/3 or 4B	0.11	-0.08	9.8×10^{-10}	3.8×10^{-3}
a_2	s_3	Utah Array	40	26	L2/3 or 4B	0.1	-0.25	1.6×10^{-12}	3.4×10^{-8}
a_2	s_4	Utah Array	49	41	L2/3 or 4B	0.13	-0.05	4.3×10^{-19}	2×10^{-2}
a_2	s_5	Utah Array	22	52	L2/3 or 4B	0.28	-0.94	3.4×10^{-49}	0
a_2	s_6	Utah Array	50	49	L2/3 or 4B	0.23	-0.16	8×10^{-59}	2.6×10^{-15}
a_3	s_1	Utah Array	42	61	L2/3 or 4B	0.05	-0.3	1×10^{-2}	6.2×10^{-19}
a_4	s_1	Neuropixels	53	190	All layers	0.04	-0.05	4×10^{-2}	3×10^{-3}
