# Supplementary material for: Relating natural image statistics to patterns of response covariability in macaque primary visual cortex
Source: Nat Commun. 2025 Jul 22;16:6757. doi: 10.1038/s41467-025-62086-1 (PMC12284261; doi:10.1038/s41467-025-62086-1)
Supplement: Supplementary file 2 — Reporting Summary [file 41467_2025_62086_MOESM2_ESM.pdf]

nature portfolio

Corresponding author(s): Ruben Coen-Cagli
Amirhossein Farzmahdi

Last updated by author(s): Jun 4, 2025

Reporting Summary

Nature Portfolio wishes to improve the reproducibility of the work that we publish. This form provides structure for consistency and transparency in reporting. For further information on Nature Portfolio policies, see our Editorial Policies and the Editorial Policy Checklist.

Statistics

For all statistical analyses, confirm that the following items are present in the figure legend, table legend, main text, or Methods section.

n/a	Confirmed	
☐	☒	The exact sample size (*n*) for each experimental group/condition, given as a discrete number and unit of measurement
☐	☒	A statement on whether measurements were taken from distinct samples or whether the same sample was measured repeatedly
☐	☒	The statistical test(s) used AND whether they are one- or two-sided<br>*Only common tests should be described solely by name; describe more complex techniques in the Methods section.*
☐	☒	A description of all covariates tested
☒	☐	A description of any assumptions or corrections, such as tests of normality and adjustment for multiple comparisons
☐	☒	A full description of the statistical parameters including central tendency (e.g. means) or other basic estimates (e.g. regression coefficient) AND variation (e.g. standard deviation) or associated estimates of uncertainty (e.g. confidence intervals)
☐	☒	For null hypothesis testing, the test statistic (e.g. F, t, r) with confidence intervals, effect sizes, degrees of freedom and P value noted<br>*Give P values as exact values whenever suitable.*
☒	☐	For Bayesian analysis, information on the choice of priors and Markov chain Monte Carlo settings
☒	☐	For hierarchical and complex designs, identification of the appropriate level for tests and full reporting of outcomes
☐	☒	Estimates of effect sizes (e.g. Cohen's *d*, Pearson's *r*), indicating how they were calculated

Our web collection on statistics for biologists contains articles on many of the points above.

Software and code

Policy information about availability of computer code

Data collection	Blackrock Microsystem and Ripple were used for collecting spike data. Expo (V1.5;https://sites.google.com/a/nyu.edu/expo/) was used to display visual stimuli and pair stimulus timings with physiological data. We recorded the Neuropixel data using SpikeGLX software and performed spike sorting with Kilosort 2.5.
Data analysis	We manually sorted waveforms using Plexon Offline Sorter (V3). Spike times and stimulus parameters were then extracted using a MATLAB script (V 2016a). Data analysis was conducted using custom code written in MATLAB 2021a (https://www.mathworks.com). Model simulations were performed using custom Python code (V3.7.11) and probabilistic programming with PyMC3 (https://github.com/pymc-devs/pymc). The code for both model simulations and data analysis is available without restrictions on GitHub https://github.com/CoenCagli-Lab/2025-NatureCommunications-Farzmahdi-et-al-code

For manuscripts utilizing custom algorithms or software that are central to the research but not yet described in published literature, software must be made available to editors and reviewers. We strongly encourage code deposition in a community repository (e.g. GitHub). See the Nature Portfolio guidelines for submitting code & software for further information.

Data

Policy information about availability of data

All manuscripts must include a data availability statement. This statement should provide the following information, where applicable:

- Accession codes, unique identifiers, or web links for publicly available datasets
- A description of any restrictions on data availability
- For clinical datasets or third party data, please ensure that the statement adheres to our policy

Data from seven sessions are publicly available through the CRCNS data sharing platform at https://crcns.org/data-sets/vc/pvc-8. Data from the remaining two sessions are available at https://doi.org/10.5281/zenodo.15596406 .The natural images used to train and test the GSM models are publicly available in the ImageNet (https://image-net.org/challenges/LSVRC/2015/) and BSDS500 databases (https://github.com/BIDS/BSDS500).

Research involving human participants, their data, or biological material

Policy information about studies with human participants or human data. See also policy information about sex, gender (identity/presentation), and sexual orientation and race, ethnicity and racism.

Reporting on sex and gender	N/A
Reporting on race, ethnicity, or other socially relevant groupings	N/A
Population characteristics	N/A
Recruitment	N/A
Ethics oversight	N/A

Note that full information on the approval of the study protocol must also be provided in the manuscript.

Field-specific reporting

Please select the one below that is the best fit for your research. If you are not sure, read the appropriate sections before making your selection.

☒ Life sciences ☐ Behavioural & social sciences ☐ Ecological, evolutionary & environmental sciences

For a reference copy of the document with all sections, see nature.com/documents/nr-reporting-summary-flat.pdf

Life sciences study design

All studies must disclose on these points even when the disclosure is negative.

Sample size	A sample size calculation was not performed; instead, the estimates are based on prior research and standard practices in the field for studying single-neuron response properties.
Data exclusions	The exclusion criteria are detailed in the Methods section under "Characterization of Neuronal Responses and Inclusion Criteria." These criteria were primarily established to confirm that neurons were visually driven, exhibited measurable responses to at least some of the presented stimuli, and had receptive fields (RFs) well-centered on the stimuli.
Replication	Reproducibility of single-neuron measurements was confirmed across different recording sessions, animals, and stimulus sets.
Randomization	Presentation of different stimulus conditions was randomized.
Blinding	N/A this study does not report a comparison between different populations.

Reporting for specific materials, systems and methods

We require information from authors about some types of materials, experimental systems and methods used in many studies. Here, indicate whether each material, system or method listed is relevant to your study. If you are not sure if a list item applies to your research, read the appropriate section before selecting a response.

Materials & experimental systems

n/a	Involved in the study
☒	☐ Antibodies
☒	☐ Eukaryotic cell lines
☒	☐ Palaeontology and archaeology
☐	☒ Animals and other organisms
☒	☐ Clinical data
☒	☐ Dual use research of concern
☒	☐ Plants

Methods

n/a	Involved in the study
☒	☐ ChIP-seq
☒	☐ Flow cytometry
☒	☐ MRI-based neuroimaging

Animals and other research organisms

Policy information about studies involving animals; ARRIVE guidelines recommended for reporting animal research, and Sex and Gender in Research

Laboratory animals	Data recorded from 4 male adult macaque monkeys (Macaca fascicularis) age 3 -8 years.
Wild animals	The study did not involve wild animals.
Reporting on sex	The study involved adult male macaque monkeys. Sex was not considered in the study design.
Field-collected samples	The study did not involve field-collected samples.
Ethics oversight	All procedures were approved by the Albert Einstein College of Medicine and followed the guidelines in the United States Public Health Service Guide for the Care and Use of Laboratory Animals.

Note that full information on the approval of the study protocol must also be provided in the manuscript.

Plants

Seed stocks	N/A
Novel plant genotypes	N/A
Authentication	N/A
